# Inferred regulons are consistent with regulator binding sequences in *E*. *coli*

**DOI:** 10.1371/journal.pcbi.1011824

**Published:** 2024-01-22

**Authors:** Sizhe Qiu, Xinlong Wan, Yueshan Liang, Cameron R. Lamoureux, Amir Akbari, Bernhard O. Palsson, Daniel C. Zielinski

**Affiliations:** 1 Department of Bioengineering, University of California San Diego, La Jolla, CA, United States of America; 2 Novo Nordisk Foundation Center for Biosustainability, Technical University of Denmark, Lyngby, Denmark; Institute for Stem Cell Science and Regenerative Medicine, INDIA

## Abstract

The transcriptional regulatory network (TRN) of *E*. *coli* consists of thousands of interactions between regulators and DNA sequences. Regulons are typically determined either from resource-intensive experimental measurement of functional binding sites, or inferred from analysis of high-throughput gene expression datasets. Recently, independent component analysis (ICA) of RNA-seq compendia has shown to be a powerful method for inferring bacterial regulons. However, it remains unclear to what extent regulons predicted by ICA structure have a biochemical basis in promoter sequences. Here, we address this question by developing machine learning models that predict inferred regulon structures in *E*. *coli* based on promoter sequence features. Models were constructed successfully (cross-validation AUROC > = 0.8) for 85% (40/47) of ICA-inferred *E*. *coli* regulons. We found that: 1) The presence of a high scoring regulator motif in the promoter region was sufficient to specify regulatory activity in 40% (19/47) of the regulons, 2) Additional features, such as DNA shape and extended motifs that can account for regulator multimeric binding, helped to specify regulon structure for the remaining 60% of regulons (28/47); 3) investigating regulons where initial machine learning models failed revealed new regulator-specific sequence features that improved model accuracy. Finally, we found that strong regulatory binding sequences underlie both the genes shared between ICA-inferred and experimental regulons as well as genes in the *E*. *coli* core pan-regulon of Fur. This work demonstrates that the structure of ICA-inferred regulons largely can be understood through the strength of regulator binding sites in promoter regions, reinforcing the utility of top-down inference for regulon discovery.

## Author summary

The Transcriptional Regulatory Network (TRN) of bacteria is a gene expression control system largely governed by transcription factors binding to promoter regions of the genome, forming a connected network of regulons. While experimental determination of functional transcription factor binding sites is laborious, inference of regulons from large-scale gene expression datasets has proven to be a powerful alternative. Here, we seek to determine whether inferred regulons have a biochemical basis in terms of binding sites sequences present at gene promoters. We use machine learning to build quantitative models relating binding site features to gene regulation. We find that the majority of data-inferred regulons can be tied to binding site sequences at gene promoters, supporting the biological reality of inferred regulons. Additionally, these inferred regulons sometimes carried additional sequence context that is not accounted for by the transcription factor binding motif extracted from experimental binding sites. An understanding of how regulons are encoded in the genome will empower sequence analysis and synthetic biology applications.

## Introduction

Microbial gene expression is tightly controlled to maintain fitness under diverse conditions. Understanding these regulatory mechanisms is critical for enabling the modification of gene expression for synthetic biology applications. Among the processes through which expression is controlled, transcription initiation stands out as a critical and highly regulated process [1]. In transcription initiation, the RNA polymerase recognizes the promoter sequence upstream of a coding region, with the polymerase sigma factor binding to a conserved motif followed by the formation of an open complex of the RNA polymerase and DNA sequence [1]. Transcription factors (TFs) often bind to promoter regions at TF-specific motifs to activate or repress transcription by altering the recruitment of the polymerase complex to the promoter. There are currently estimated to be over 200 TFs and 7 sigma factors in *E*. *coli*, for which an increasing fraction have characterized regulons [2]. This set of regulons constitutes the transcriptional regulatory network (TRN) of *E*. *coli*, representing thousands of interactions between regulators and promoter sequences [2]. While our understanding of the TRN of *E*. *coli* likely exceeds that of any other organism, there is still much that we do not understand regarding how the TRN is encoded in the genome itself [3,4].

A variety of experimental and computational approaches have contributed to our current knowledge of the structure of the TRN of *E*. *coli*. Chromatin immunoprecipitation (ChIP) is a bottom-up method for TRN elucidation that identifies TF binding sites across the genome by crosslinking bound proteins to DNA and then sequencing the captured DNA segments [5]. These binding sites are measured with particular signal-to-noise (S/N) ratios, depending on the number of reads of a given sequence present after alignment. As ChIP experiments alone do not indicate whether the TF binding regulates expression of the gene, ChIP is often paired with differential expression analysis after TF knockout to validate that the TF binding is regulatory [6]. A set of binding sites above a certain S/N enrichment threshold that also affect gene expression comprise a bottom-up, component-by-component estimation of the TF regulon. Over the years, this process has been repeated for many of the major *E*. *coli* transcription factors, enabling a bottom-up, ChIP-based estimation of the TRN of *E. coli [7,8]*.

As an alternative approach, network inference methods have been used to provide top-down estimates of the TRN based on analysis of RNA-seq compendia [9]. In particular, independent component analysis (ICA) of a large *E*. *coli* RNA-seq dataset has recently provided a highly informative estimation of the TRN of *E. coli [10]*. ICA is an unsupervised machine learning method that identifies statistically independent sets of coordinated variables, termed components [11]. When applied to gene expression data, ICA identifies sets of genes that display similar expression patterns, while minimizing overlap in gene content of different sets. ICA on a data matrix **X** results in two matrices: **M** that contains the gene weights in each component (to which a threshold is applied to establish component composition), and **A** that contains the activity level of each component on specific experimental conditions. ICA has been applied to large RNA-seq expression compendia to identify independently modulated groups of genes, termed iModulons, in multiple organisms [12]. These ICA-calculated regulons often overlap significantly with ChIP-determined regulons, supporting the biological significance of ICA-calculated TRNs. However, it is still unclear whether a biochemical basis for these regulons can be found that specifies their gene membership, for example in terms of regulator binding sequences found uniquely at these promoter regions.

In this study, we developed a quantitative understanding of the *E*. *coli* TRN by constructing machine learning models that predict ICA-inferred regulon membership based on promoter sequence. With these models, we identified important features that determine regulon membership. We utilized these machine learning models to understand the basis for complex top-down regulons involving multiple regulators. Further, we investigated differences between bottom-up and top-down TRNs to find the general factors underlying TRN estimates from these methods. Furthermore, we expanded the scope of our analysis to multiple strains of *E*. *coli*, and we explained variation of regulatory activity across strains using sequence features.

## Results

### Promoter sequence features can quantitatively predict a large part of the *E*. *coli* TRN

**Fig 1:**
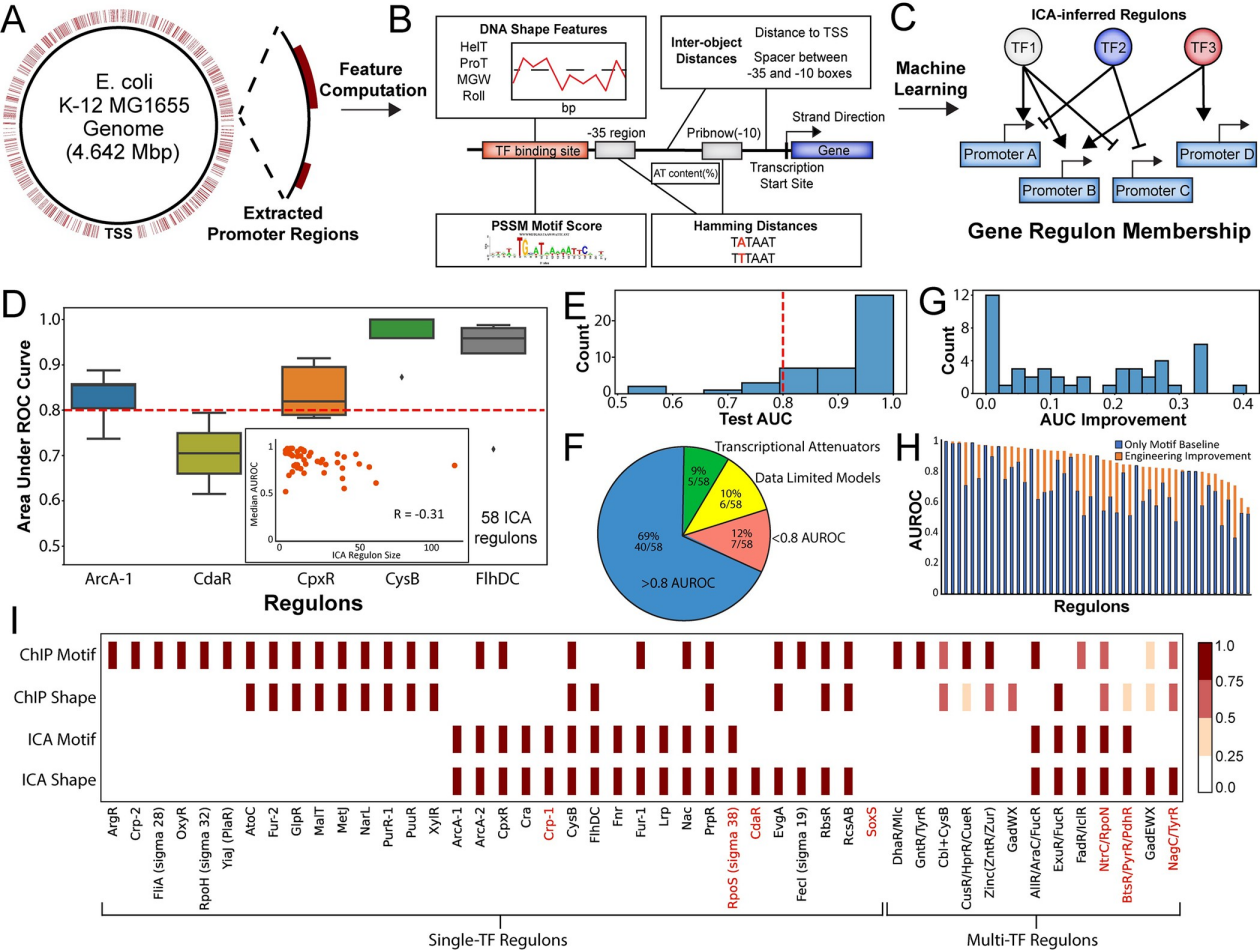
Sequence-based TRN prediction workflow and model assessment. (A) Gene promoter regions are extracted from the genome sequence of *E*. *coli* K12 MG1655. (B) Feature computation from the promoter sequence extracted from the genome. (C) Feature matrix organization and ICA regulon membership as the target label. (D) Logistic regression classifier model assessment by area under receiver operator characteristic (ROC) curve and overall predictive model status. Cutoff = 0.8 for good models. (E) Histogram of Area under the ROC curve for all 58 models. (F) Overview of model performance of good (AUC > 0.8), bad (AUC < 0.8), data limited models, or regulons that could not be modeled due to a transcriptional attenuation regulatory mechanism. (G) Histogram of the improvement between motif-only models and models trained with engineered features. (H) Bar chart showing the performance of motif-only models and models trained with engineered features. (I) Overview of feature importance among the top 5 features in each model. For multi-transcription factor regulons, the shading indicates how many of the enriched regulators appeared among the top 5 features in the model for the regulon. Red text indicates a bad performing model (AUC < 0.8).

First, we constructed machine learning classifiers to predict gene membership in ICA regulons using promoter sequence features. ICA regulons (also called iModulons) are sets of genes that show similar expression patterns. We developed a workflow to quantify the gene promoter sequence into a sequence feature matrix, and then trained a logistic regression (LR) classifier to predict ICA regulon membership (**Fig 1A–1C**). We note that we also tested other machine learning model types, including support vector machines and random forests, and found that logistic regression broadly exhibited the least amount of overfitting.

The feature matrix used contains: 1) sigma factor related features, including motif score and Hamming distance of -10/-35 boxes that comprise the RNA polymerase binding site, spacer length between the -10/-35 boxes, AT content (**Fig A in S1 Text**), 2) TF related features, including motif score and shape features (**Fig B in S1 Text**), and 3) genome organization features, including strand direction and binding site distance to transcription start site (**Fig 1B**). The sequence features were calculated for all 7 sigma factors, 59 TFs and ICA regulon motifs. For feature engineering, linear discriminant analysis (LDA) transformation was applied to sigma factor related features and 14 DNA shape features (see Methods). We included TF binding site motifs derived from both ChIP and ICA regulons, to investigate any differences in sequence context that may underlie differences in these regulon sources. We term the full set of features as ‘engineered features’, the performance of which we contrast with the use of the TF motif scores as the only features. The detailed information of total 204 features can be found in the project Github Repository at https://github.com/SBRG/IM-ML/.

The current version of the *E*. *coli* K12 MG1655 TRN estimated by ICA contains 58 total regulons [13]. Models could not be constructed for 6 of these regulons due to small size (only containing a single promoter) or poor promoter annotation, while 5 of the regulons were regulated by transcription attenuation that is not encoded in the promoter. Model assessment by cross-validation showed that models for 40 of the remaining 47 regulons had area under receiver operating characteristic curve (AUROC) scores exceeding a 0.8 threshold, while models for 7 regulons performed under this threshold (**Figs 1D-1F and 1C in S1 Text**). We additionally compared models constructed using ChIP-defined motifs only with models constructed with the engineered features (the full set of features), and found that the median improvement in AUROC was 0.15 (**Fig 1G and 1H**).

By examining the resulting machine learning models, we then determined the most essential sequence features for defining regulon membership (**Fig 1I**). We examined the top 5 features of the LR models as ranked by SHAP values [14]. Models for 6 regulons showed good performance with only ChIP motif information required. The remaining models required additional sequence features, such as alternate ICA regulon-specific motifs or DNA shape features. Other features such as -10/-35 box information or spacer information were not highly weighted in any regulon models. SoxS was the only regulon that showed no regulator-related features among the top 5 features, consistent with the poor performance of its model.

**Fig 2:**
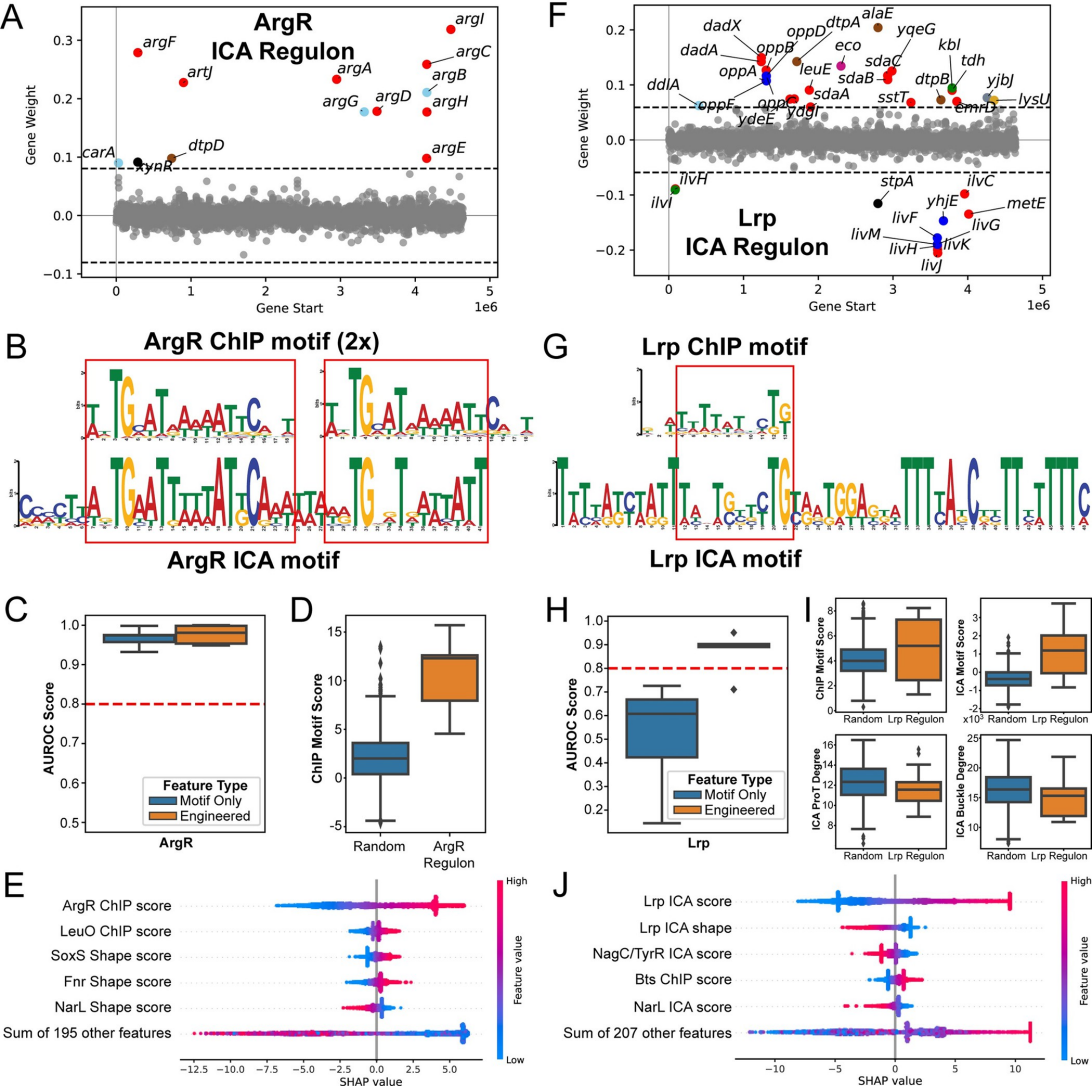
Examples of predictive models along with regulon-determining sequence features. (A) ArgR regulon determined by ICA mapped across the E. coli genome (B) ChIP and ICA regulon motifs of ArgR. (C) Model performance of argR with only the motif vs with engineered sequence features. (D) ArgR motif strength in ArgR-regulated promoters vs random promoters. (E) Feature importance for the ArgR model. (F) Lrp regulon determined by ICA mapped across the E. coli genome (G) ChIP and ICA regulon motifs of Lrp. (H) Model performance of Lrp with only the motif vs with engineered sequence features. (I) Lrp motif strength and shape features in Lrp-regulated promoters vs random promoters. (J) Feature importance for the Lrp model.

We discuss two representative examples of highly accurate models, for ArgR and Lrp (**Fig 2**). The ArgR regulon is an example of the ChIP motif being sufficient to define the regulon membership across the genome. The ArgR regulon consists of mostly arginine biosynthetic genes that are spread out spatially across the genome (**Fig 2A**). The ChIP and ICA-determined motifs for ArgR have significant apparent overlap, with the ICA motif containing two homologous regions to the ChIP motif (**Fig 2B**). Consistent with this agreement, the ChIP motif alone already performs well in specifying the ArgR regulon (**Fig 2C**), with genes within the regulon having much higher motif scores than genes outside the regulon (**Fig 2D**). Correspondingly, the resulting machine learning model weights the ChIP motif as by far the most important feature (**Fig 2E**). The dimer motif noted within the ICA motif underlies a well-characterized hexamer structure of ArgR-DNA complexes [15], thus it is surprising that the strength of a single ArgR ChIP motif is sufficient to specify a functional binding site. It is possible that a strong primary motif is predominantly responsible for recruiting the complex to the binding site, while the surrounding motifs serve a supporting or stabilizing role.

The Lrp regulon is an example where the ChIP motif was insufficient to build a predictive model, but additional engineered sequence features enabled successful determination of a predictive model. Similar to ArgR, Lrp is an amino acid regulator that regulates diverse genes located spatially across the genome (**Fig 2F**). Unlike ArgR however, while the ChIP and ICA motifs for Lrp have a homologous region, the ICA motif contains an additional large consensus sequence (**Fig 2G**). Reflecting this, a model constructed with only the Lrp ChIP motif performed poorly, while a model consisting of additional engineered sequence features was able to successfully predict Lrp regulon membership (**Fig 2H**). These additional features included the ICA motif as well as shape features, which are statistically differential (p<10^−2^, Mann-Whitney U test) between the Lrp regulon and random genes (**Fig 2I**). SHAP values verify that regulator binding site motif scores and shape features are important features (**Fig 2J**). The ICA-calculated extended motif does not clearly contain a repeated sequence, so the significance of these regions is still unclear. However, we note that the motif is derived from diverse promoters (**Fig 2F**) and has highly consistent locations outside of the ChIP motif region, most notably an extended multi-T region.

### Models can be improved through TF-specific sequence features

**Fig 3:**
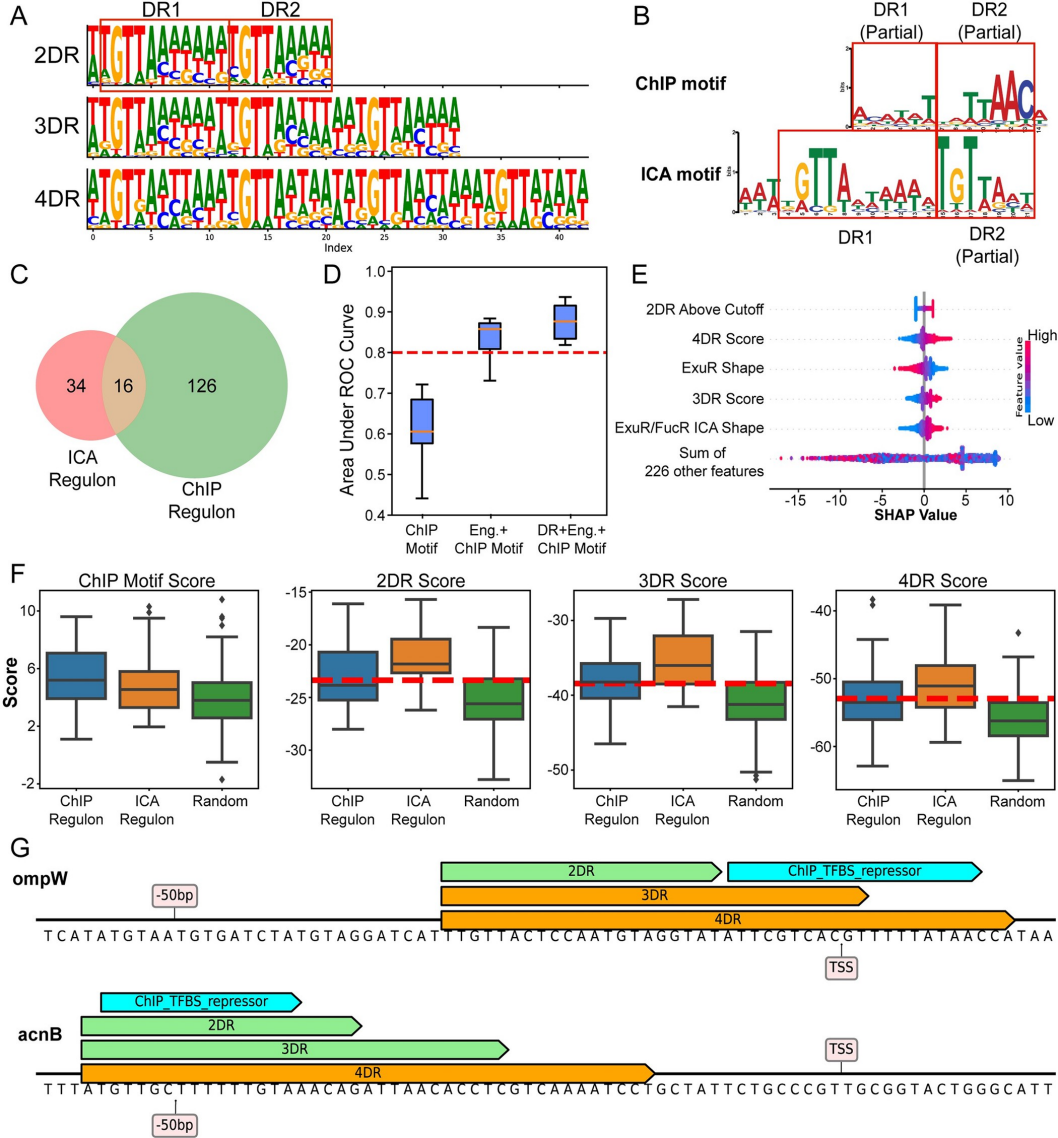
Regulator-specific features improve model performance for ArcA regulon prediction. (A) Direct repeat motifs previously defined for ArcA. (B) ChIP and ICA motif of ArcA with direct repeats annotated. The expected direct repeat motif appears stronger in the ICA motif. (C) Venn diagram of ArcA ChIP and ICA regulons, showing the number of genes in each as well as the overlapping set. (D) Comparison of prediction accuracy with original feature matrix and multiple DR features. (E) Feature importance of ArcA ICA regulon prediction assessed by SHAP values. (F) Distributions of multiple DR motif scores and ChIP motif scores for ICA/ChIP regulons and random sequences. (G) Multiple DR motifs aligning to promoter sequences. Green: motif score above cutoff, Orange: motif score below cutoff, Blue: experimentally determined binding sites. Cutoff values are average motif scores of confirmed DR motifs.

ArcA is an important global regulator involved in redox regulation [6]. The initial machine learning model for ArcA showed relatively poor performance, indicating that features we used, which included the ArcA binding motif determined by ChIP, were not sufficient to determine the ICA regulon membership. It has been proposed that the binding site of ArcA is quite diverse and involves a variable number of direct repeats (DRs) of the ArcA motif [16]. Thus, rather than calculating a single ArcA motif score, we hypothesized that it would be more biochemically accurate to define scores for multiple possible DR motifs with letter probability matrices (**Fig 3A**). Interestingly, the ChIP and ICA motifs both contain a partial representation of this direct repeat structure but fail to capture complete repeats (**Fig 3B**). We found that the overlap between ICA and ChIP-determined regulons was relatively low (**Fig 3C**), which likely explains the differences in their corresponding motifs. Using position-specific scoring matrices (PSSMs) generated from letter probability matrices, we calculated DR motif scores and added these features to the ArcA model. Comparing predictive models generated using multiple DR motifs and those using only ChIP motifs separately, we found that ArcA model performance was greatly improved by the inclusion of DR motif scores (**Fig 3D**). Additionally, the model feature weights suggest that these direct repeats are critical to predicting ArcA regulon membership (**Fig 3E**). The lower distributions of 3DR and 4DR motif scores in ArcA ChIP regulon suggest that it fails to capture genes with promoters containing extended DR elements, and the ICA regulon is a good supplement to the missing information (**Fig 3F**). Example promoters demonstrate how the DR motifs align with experimentally determined ArcA binding sites (**Fig 3G**). With better understanding of regulatory interactions, more useful features like the DR motifs of ArcA can be extracted from the sequence to explain regulons and improve poor performing machine learning models.

### Consensus regulons across TRN-estimation methods contain strong sequence features

**Fig 4:**
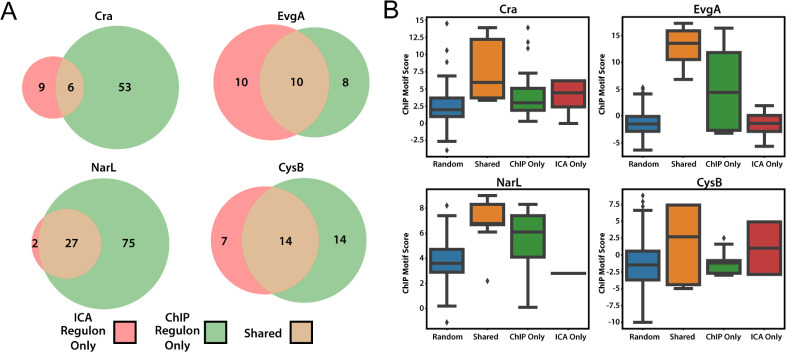
Comparison between ICA and ChIP-estimated regulons. (A) Venn diagrams showing shared (brown) and unique (red/green) regulons estimated by each method. (B) Comparison of ChIP motif scores in the consensus regulon and unique regulons. Genes in the shared regulons display a markedly higher distribution of motif scores.

Noting substantial differences between ICA and ChIP-estimated regulons for certain regulators, we wanted to determine whether promoter sequence features underlie any of the observed differences between ICA and ChIP-estimated regulons. We compared the promoter features of the genes within ICA and ChIP estimations of six single TF-associated regulons (**Fig 4A**). Motif scores of genes in the overlapping region of ChIP and ICA regulons were found to be significantly higher than those of the remaining genes in the unique regulons (**Fig 4B**). Thus, the genes that are predicted to be in a regulon by multiple methods are likely the highest confidence functional binding sites. Additionally, in three of the four observed cases, EvgA, NarL, and CysB, the non-overlapping regulons had substantially different motif scores between genes in the ChIP and ICA regulons. This could suggest that the regulons could contain different sub-motifs that are captured differently by each method.

### The core Fur pan-regulon contains stronger sequence features and greater binding strength

**Fig 5:**
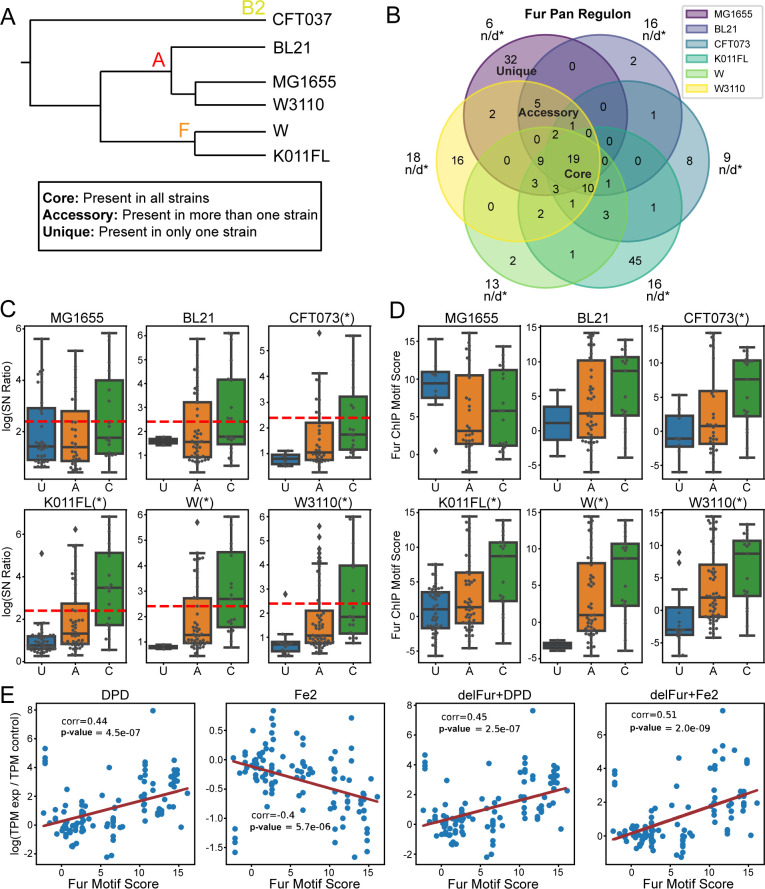
Fur pan-regulon analysis. (A) The phylogenetic tree of 6 strains investigated. (B) Fur pan-regulons for transcription units for 6 strains, with the phylogenetic tree displayed and unique, accessory, core regulons annotated. (C) Comparison of S/N ratios and Fur motif score: core > accessory > unique. (D) Expression change($log(\frac{tpm_{experiment}}{tpm_{control}})$) versus Fur motif score. Regulatory response correlates well with the motif score.

To explore TRN variation across multiple *E*. *coli* strains, we reconstructed the Fur pan-regulon of genes and transcription units for 6 strains across 3 phylogroups (**Figs 5 and D in S1 Text**). The S/N ratios of Fur binding peaks from ChiP experiments in the core regulon were compared (**Fig E in S1 Text**). Strains W and KO11FL in the F phylogeny group have higher Fur binding peak S/N ratios compared to MG1655, while CFT073 in B2 group has relatively low S/N ratios, indicating lower regulatory activity. Strains in the same phylogroup tend to have similar regulatory activity.

Genes with ChIP-exo peaks in promoters are grouped into those with high and low S/N ratios at a cutoff of 10 (**Fig F in S1 Text**). While genes of low S/N ratios overlap little with Fur ChIP/ICA regulons, genes of high S/N ratios have large overlap with both types of regulons (**Fig G in S1 Text**). In addition, Fur ICA regulons determined with MG1655 RNA-seq expression data appear robust at capturing genes of high S/N ratios across multiple strains (**Fig H in S1 Text**), which is consistent with the finding in the previous section that the ICA-based TRN contains genes of strong regulatory activity.

In the Fur pan-regulon, only genes in the core regulon are regulated in each strain; unique and accessory regulons vary across strains. Fur regulatory activity tends to be highest for core regulon genes, followed by the accessory regulon, and finally by the unique regulon. Motif scores follow a similar trend. These results suggest that binding site strength is the major factor causing regulatory activity variation between the core, accessory, and unique regulons (**Fig 5C**). Conserved promoters across strains tend to have high binding site strength, and thus high regulatory activity. Furthermore, a higher motif score indicates a stronger regulatory response, characterized by the expression change between control and experiment conditions (**Fig 5D**). These tendencies further validate that motif score is a good indicator of regulatory activity. In summary, the sequence basis of TRN’s variation across strains lies in binding site strength, characterized by motif scores.

## Discussion

We developed DNA sequence-based machine learning models for gene regulon membership, resulting in high prediction performance for 40/47 of possible *E*. *coli* ICA regulons. Successful models highlight that gene transcriptional regulation can be quantitatively explained by relatively simple promoter sequence features for many regulons. We found that TF motif scoring and DNA shape are both critical for determination of top-down or bottom-up regulon membership. However, certain regulons require additional specialized transcription factor-specific sequence features, exemplified by the need to add ArcA direct repeat motifs to obtain a good model for the ArcA regulon.

Generally, a higher motif score indicates higher similarity to the consensus sequence, but it only accounts for binding site strength with positional frequency, leaving out other properties of DNA sequences. Therefore, the motif score is important but not always sufficient to determine regulon membership. To characterize regulator-sequence interactions structurally, DNA shape features were included in the feature matrix for machine learning models, and they acted as significant features in the prediction, consistent with previous work in this area [3]. TF-specific DNA shape vectors reflect unique regulator protein structures and provide another measurement for regulator binding affinity.

We further note that ICA regulons were used to derive TF motifs in addition to the ChIP-derived motifs. ICA-derived motifs create a partially circular workflow when used as features in machine learning to predict ICA regulon membership. Still, the workflow is not entirely circular because ICA only defines the positive set of genes (those expected to have the binding site motif), but not the negative set (promoters that are not regulated by the TF). We justify the inclusion of ICA-derived motifs as machine learning features with the purpose of determining whether the motif derived from the positive ICA gene set is sufficient to distinguish promoters that are regulated by the TF from those that are not. A second advantage to the analysis is that we can directly analyze the difference between the ChIP and ICA motifs, to understand what sequence features underlie the more functional binding site extracted from ICA. This for example revealed the importance of the direct repeat motif for ArcA, which was stronger in the ICA-derived motif than the ChIP-derived motif.

Comparing ChIP and ICA regulons demonstrates that ICA seems to preferentially identify genes of strong binding site strength. If a gene is included by both types of regulons, the regulatory interaction is not only assessed by ChIP based experiments but also validated by unraveled sources of transcription signals. Genes unique to ICA regulons also have high motif scores, indicating potentially undetected parts of the TRN. Similarly, binding site strength and S/N ratios also distribute unevenly across unique, accessory and core regions in the Fur pan-regulon: the core regulon is more active than the unique regulon due to high conservation level of promoters in core regulon. Considering the relatively larger overlap between high S/N ratio sites and two types of regulons, the S/N ratio might also be an indicator of the confidence level, in which case, the regulatory interactions in core regulon have a higher confidence level.

There is substantial room for improvement of model performance for poorly performing regulons. Models for 6 ICA regulons could not be determined due to lack of necessary information for feature computation, such as regulator binding site motifs for small regulons or transcription start site annotation. Improvements on sequence feature computation will improve the accuracy of machine learning and thus more top-down regulons would be explainable. A better motif matching method than linear search with PSSM could be developed to improve the computation of motif scores and better characterize binding site strength. Though PSSM provides the probability of the segment being a binding site, the shape of the DNA sequence is not considered. There have already been a few studies using DNA shape in binding site prediction, for example, improvement was observed in the machine learning model using both DNA sequence and shape to predict TF binding sites in ChIP-seq datasets [17]. Therefore, predicting a TRN will become more accurate if the scoring function used in motif matching can be formulated with both PSSM and DNA shape information. In addition, further characterization of TF-specific sequence features could improve prediction of regulons with poorly performing models. For instance, multiple direct repeats motifs of ArcA characterize diverse binding site architecture better than the ArcA original ChIP-determined motif, and inclusion of this ArcA-specific sequence feature improves the prediction accuracy for the ArcA ICA regulon. Given enough data, the machine learning workflow should be able to reconstruct the TRN with high accuracy.

Although we use simple machine learning models used in this work, there has been substantial progress in recent years building deep learning models to predict the function of non-coding regions of DNA [18,19]. One advantage of our approach is that it yields reasonable results with less data. This is the advantage of feature engineering, while the drawback is reduced power compared to end-to-end deep learning for large datasets that can create its own features. We expect that in the future, there will be hybrid models that take advantage of the power of deep learning as well as biological regularization such as motif binding scores that take advantage of existing knowledge of regulatory mechanisms.

Taken together, this study introduces a reliable workflow to analyze transcriptional regulation activity in prokaryotes based on promoter sequence, using machine learning models to reconstruct the TRN. The requirements for this workflow are inferred regulons, promoter architectures of the organism, and transcription factor binding site motifs. While this data is most available in model organisms in *E*. *coli*, it should be possible to extend this to other organisms with minimal additional experimentation. For example, we have recently demonstrated that the regulon machine learning models can be constructed for certain regulons in *Limosilactobacillus reuteri* [20], showing its applicability across bacteria. Biophysical factors affecting TRN architecture and regulatory activity were surveyed, offering directions for data-driven genome design in synthetic biology to control cellular activity by tuning sequence features.

## Methods

### The Bitome: Curating and organizing genomic feature information

Genome information for *Escherichia coli* K-12 MG1655 was collected and curated from the NCBI RefSeq and RegulonDB databases [21]. The Bitome Python software package was utilized to load and organize sequence-based information from these data sources, including genome sequence, coding sequences, transcription start sites, transcription units, and transcription factor binding sites [22]. The code containing workflows used in this work is available at https://github.com/sbrg/IM-ML.

### Motif score of promoter sequence

A position-specific scoring matrix, or PSSM, was generated for a given TF by aligning its known binding site sequences and computing the relative probability of finding each DNA base at each binding site position. As the PSSM coefficients are the log-odds of a probability, the summation of the log-odds at all nucleotide positions in a putative binding site represents the probability of motif existence. The algorithm performs 1-D dynamic programming that iteratively computes the motif score of consecutive equal-length segments (length = motif length), and the segment of highest score is the matched motif box. Then, the highest score is output as the motif score of the promoter sequence.

For transcription factors, the search range was -150bp and +50bp to the transcription start site, and in this study, PSSMs were obtained from RegulonDB’s TF PWMs Browser page, mainly determined experimentally by ChIP seq experiments [21]. Unlike TF motifs, sigma factor motifs consist of two binding regions with a gap of varying length: the -10 box (Pribnow box) and -35 box. PSSMs of -10 and -35 boxes for sigma 70/32/38/24/54/28 were generated from annotated sigma factor binding sites in RegulonDB and consensus sequences from the reference textbook *Transcription regulation in prokaryotes [23]*. Other sigma factors were not included due to limited annotation. The search range was 20bp upstream for -10 box, 40bp for -35 box. In addition to motif scores of -10/-35 boxes on promoter sequences, an AT-rich 7bp segment in the spacer between -10 and -35 boxes was matched and the highest AT ratio was used to account for the extended -10 box [24]. Sigma factor related features were motif scores and Hamming distances of -10/-35 boxes, the distance from -10/-35 boxes to the transcription start site, spacer length, AT ratio of the spacer, and AT ratio of the extended -10 box. To reduce feature dimensions, a hyperplane decision surface, dividing the sigmulon (genes regulated by the sigma factor) and other genes, was computed using linear discriminant analysis (LDA), and features were projected to it.

Some motifs in RegulonDB failed to capture the whole binding site consensus sequence, or other motifs also important to regulation activity were missed. For example, the Fur binding site motif in RegulonDB didn’t include the whole 19bp length inverted complementary repeats structure [25]. Motif discovery in ICA regulons using the MEME suite provided motifs missed by RegulonDB, named “ICA motifs” in contrast to “ChIP motifs.” MEME is a toolbox used to discover novel motifs in collections of unaligned sequences [26]. Those ICA regulon motifs were used to supplement information missed by ChIP experiments.

Another possible edge case was that the regulator binding site’s architecture has great diversity, which could not be characterized by a simple motif. For instance, 42% of ArcA binding sites are 2 direct repeats of 10bp segments, 41% are 3 direct repeats, 15% are 4 direct repeats and 5% are 5 direct repeats [16]. And hence, direct repeats (DR) motifs were used to compute features for model performance improvement.

### DNA shape feature computation

In this study, 13 types of shape features were computed using DNAShapeR, a DNA shape predictor in R language [27]. These shape features included 6 inter-base pair shapes: Roll, Helix Twist (HelT), Shift, Slide, Rise and Tilt, and 7 intra-base pair shapes: Minor Groove Width (MGW), Propeller Twist (ProT), Buckle, Shear, Stretch, Stagger and Opening.

A pentamer query table integrated with a sliding-pentamer window was used to compute the shape vectors for all matched TF binding sites. The pentamer table was obtained from the GitHub repository of DNAShapeR. For predicting intra-base pair parameters, each sliding step assigned a shape prediction for the central base pair. For predicting inter-base pair parameters, each sliding step assigned a shape prediction for two central base-pair steps. The overlapping values arising from two adjacent pentamers at the same nucleotide position were averaged. The sliding-window approach thus results in a shape vector [28]. Maximum, minimum, range, and mean values of the shape vector were computed as features. To reduce dimensions of shape features, LDA was applied to project shape features onto a hyperplane decision surface, which divides the ChIP regulon and other genes.

### Logistic regression classification on ICA regulon membership and assessment of model performance

A logistic regression classifier was chosen in this study because it is a more interpretable model compared to common alternatives such as Random Forest or Neural Network models, and the degree of overfitting is usually smaller. The classifier was implemented using “sklearn.linear_model.LogisticRegression” in the scikit-learn Python package [29]. A default elastic net penalty with 0.5 L1 to L2 ratio was used for a combination of L1 and L2 regularization. The target labels were binarized ICA regulon memberships. Two feature matrices were trialed: only ChIP motif scores, and all features. Models that had more than five promoters were assessed by 5-fold cross-validation with stratified sampling preserving the percentage of samples for each class. Cross-validation was implemented as follows: The dataset was divided into 5 splits, and a model was trained with fixed hyperparameters on each split with 80% of the data, testing on the remaining 20%, using the default value for the elastic net regularization weighting. An average test performance value (AUROC) was calculated to assess model performance. For models smaller than 5 promoters, n-fold cross-validation was performed where n equals to the number of promoters. The test was performed in promoter space where genes from the same promoter were kept in the same group during the random split of the entire dataset into a train set and a test set. SMOTETomek resampling with k_neighbors = 5 was implemented using the Imbalanced-learn Python package [30] to achieve a balanced split of train and test sets. For models containing less than five genes, random oversampling was used to boost the positive samples to five. Area under the curve of receiver operating characteristic (AUC ROC) was used to evaluate performance, showing true positive rate versus false positive rate across all possible classification thresholds. A cutoff of 0.8 was used to indicate good performance.

### Promoter prediction and pan-regulon reconstruction

Though genomic information of *E*. *coli* MG1655 is well annotated, other strains, such as W3110, have limited genomic objects annotated, especially the lack of transcription start sites that indicate the location of promoters. Therefore, predicting promoter locations was necessary for the multi-strain study of *E*. *coli*. We aligned sequences of transcription units in MG1655 to other strains’ genome sequences using BLAST [31]. The other strains studied were: KO11FL, CFT017, W, W3110, and BL21. Inexact alignments were filtered out; only exact matches were used to map MG1655 transcription units onto the genomes of other strains. In this manner, transcription start sites were also determined for all strains.

After promoters were predicted for each strain, Fur ChIP-exo S/N ratios were mapped to promoter sequences, and transcription units regulated by Fur in each strain were determined. Since predicted transcription units were all exact matches, the genes in transcription units were known, therefore genes regulated by Fur were also determined. The Fur pan-regulons of genes and transcription units was then reconstructed for the six strains of *E*. *coli*: MG1655, KO11FL, CFT017, W, W3110 and BL21, across three phylogroups: A, B2 and F. Unique, accessory and core regulons were annotated based on shared Fur regulation across strains.

## Supporting information

S1 Text Contains Figs A-H. (DOCX)

S1 Data Contains tables of regulon content, promoter data, transcription factor motifs, and model results. (XLSX)
