# Supplementary material for: Inferred regulons are consistent with regulator binding sequences in E. coli
Source: PLoS Comput Biol. 2024 Jan 22;20(1):e1011824. doi: 10.1371/journal.pcbi.1011824 (PMC10833566; doi:10.1371/journal.pcbi.1011824)
Supplement: S1 Text — (DOCX) [file pcbi.1011824.s001.docx]

**Supplementary Information for**

Inferred regulons are consistent with regulator binding sequences in E. coli

Sizhe Qiu, Xinlong Wan, Yueshan Liang, Cameron Lamoureux, Amir Akbari, Bernhard O. Palsson, Daniel C. Zielinski

Daniel C. Zielinski

Email: [dczielin@ucsd.edu](mailto:dczielin@ucsd.edu)

**This PDF file includes:**

Supplementary Figures


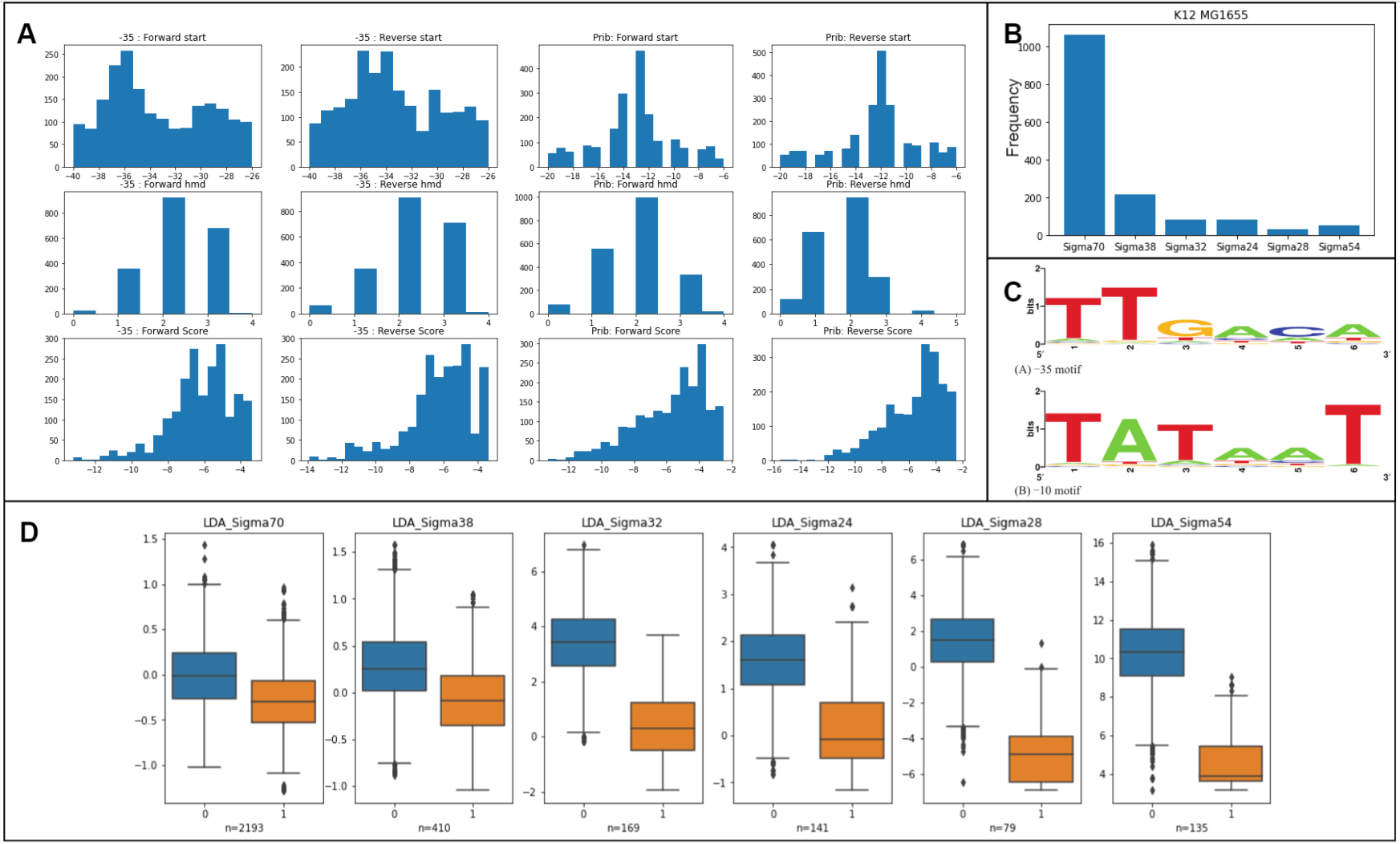


**Fig A.** Sigma factor binding boxes matching results. (A) Locations, hamming distances and motif scores of sigma factor 70’s matched Pribnow (-10) box and -35 box. Consistent results for genes in reverse and forward strands. (B) The sizes of experimentally determined sigmulons: sigma factor 70/38/32/24/28/54. (C) Motifs of sigma factor 70’s -10 and -35 boxes. (D) Distribution of sigma factor related features processed by LDA. 1 for genes in the sigmulon and 0 for other genes. Features include distances of -10/-35 boxes to transcription start sites, motif scores, Hamming distances, spacer length, AT content of extended -10 box in the spacer.


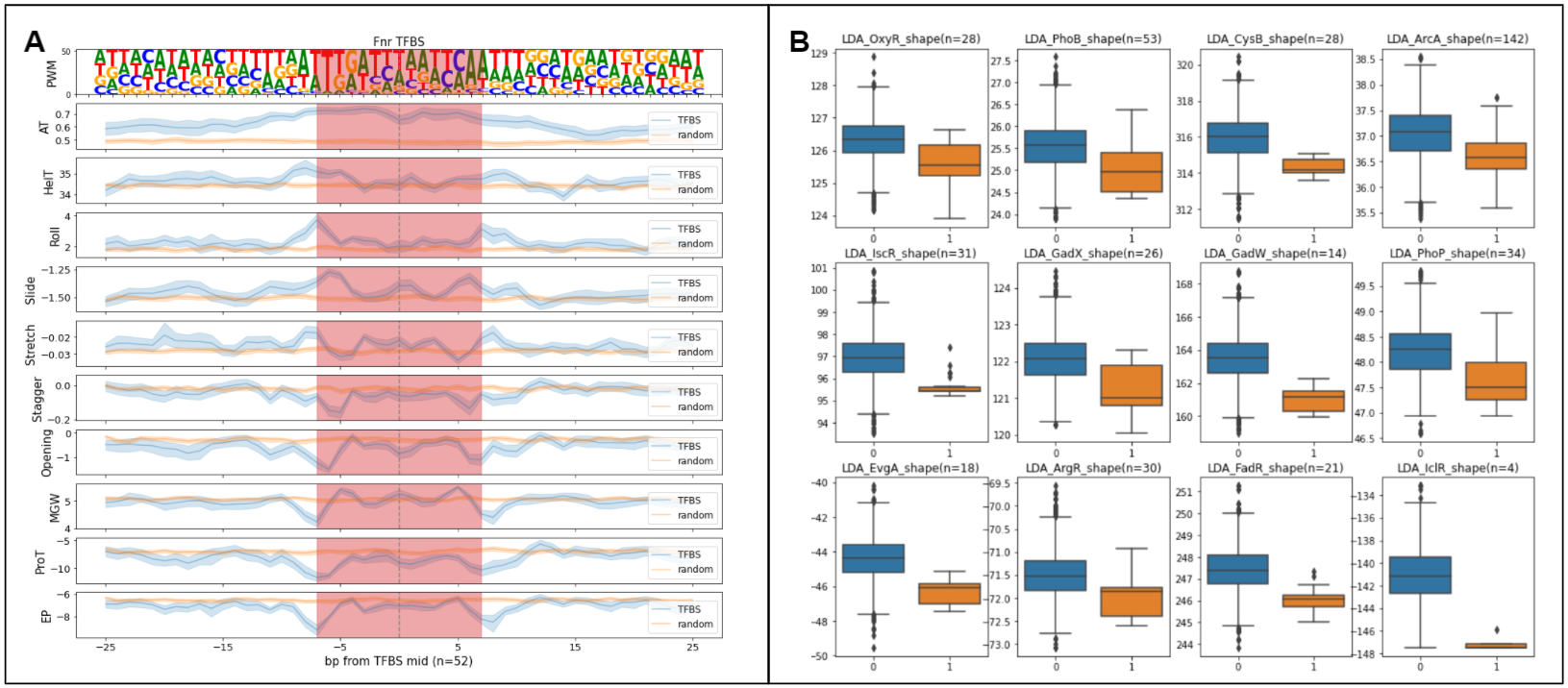


**Fig B.** DNA shape profile. (A) DNA shapes at Fnr TFBS. Shapes include HelT, MGW, ProT, roll, slide, stretch, stagger and opening. (B)Distribution of DNA shape features transformed by LDA. 1 for genes in ChIP regulon of the TF, and 0 for other genes.


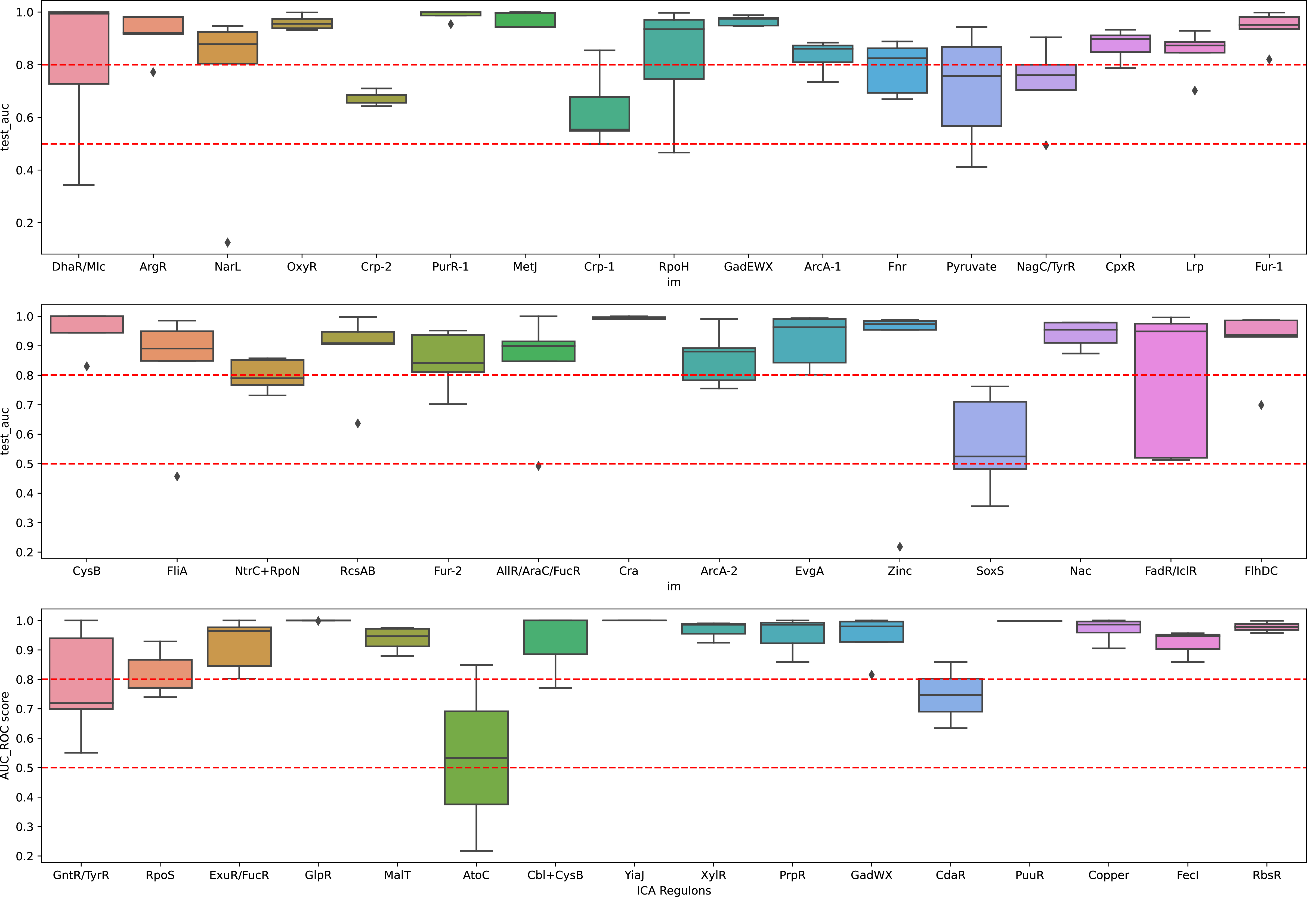


**Fig C.** Model assessment for 47 ICA regulons. The 0.5 AUC ROC is a reference point for completely random models, and 0.8 is a threshold for good models.


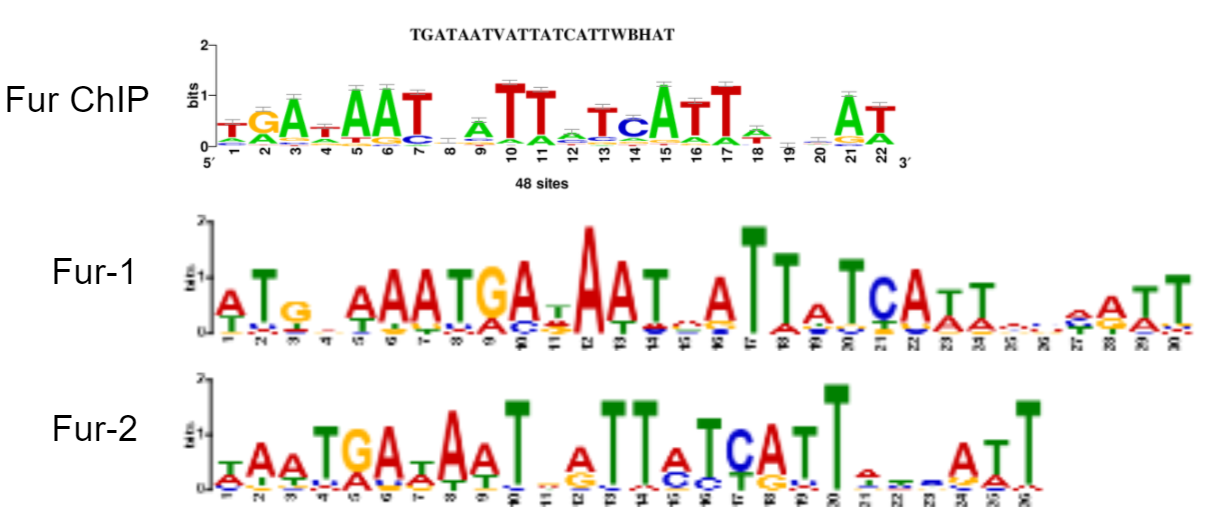


**Fig D.** ChIP and ICA regulon motifs of Fur in E.coli K-12 MG1655.


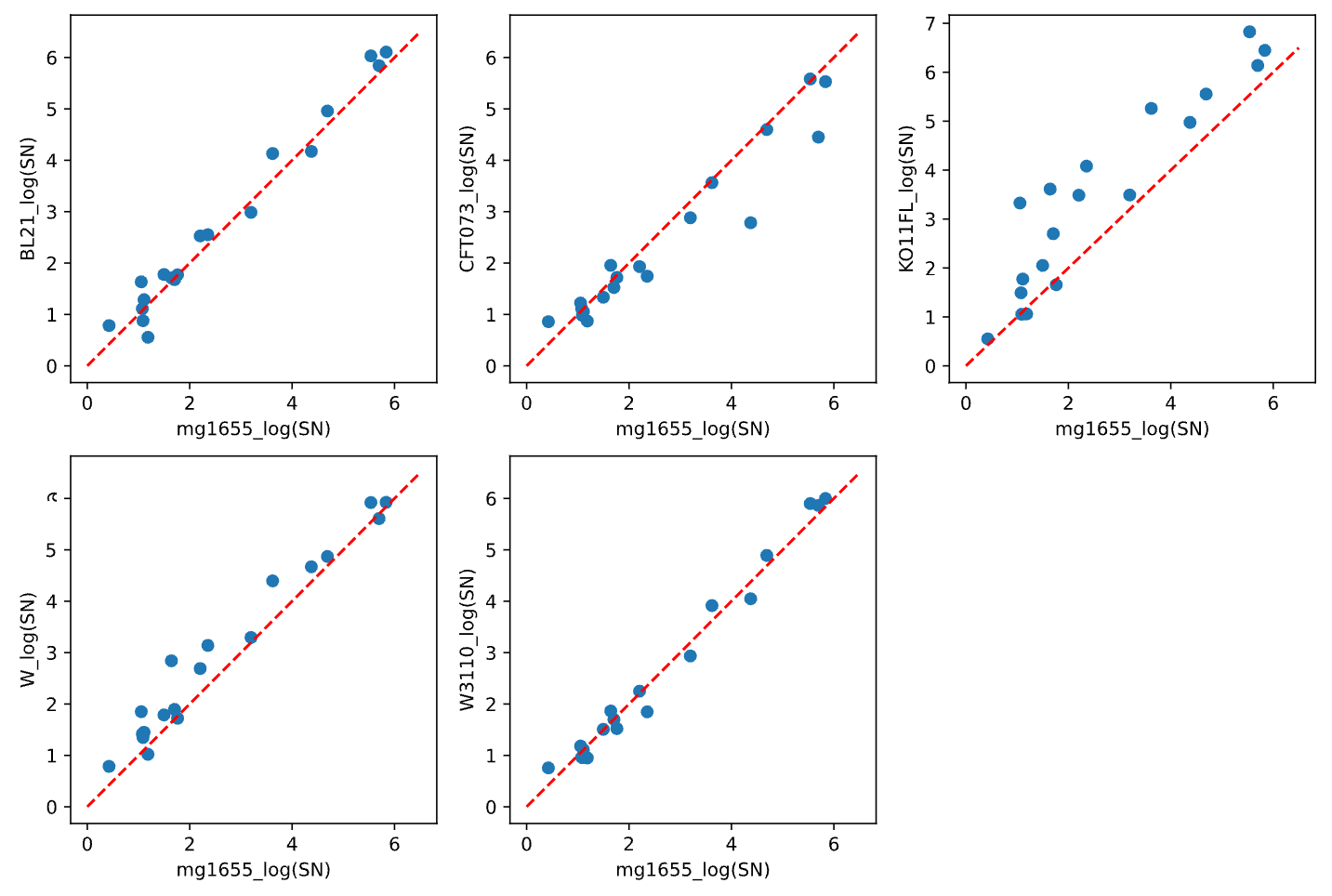


**Fig E.** Comparison of S/N ratios between MG1655 and all other 5 strains.


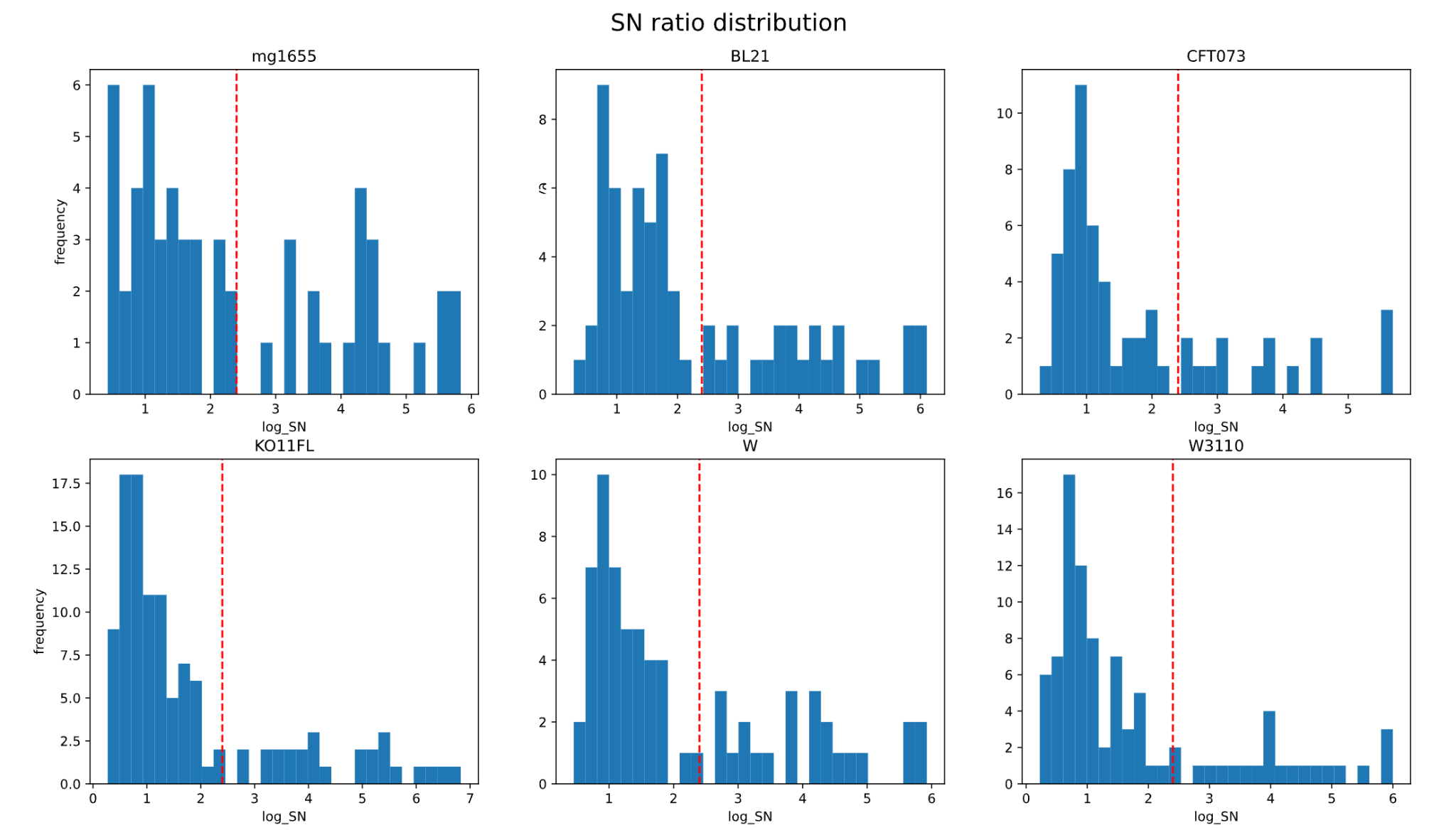


**Fig F.** Histograms of S/N ratios in each strain. Bimodal distribution can be observed and a cutoff = 10 (2.3 in log-scale) separates high and low S/N ratios.


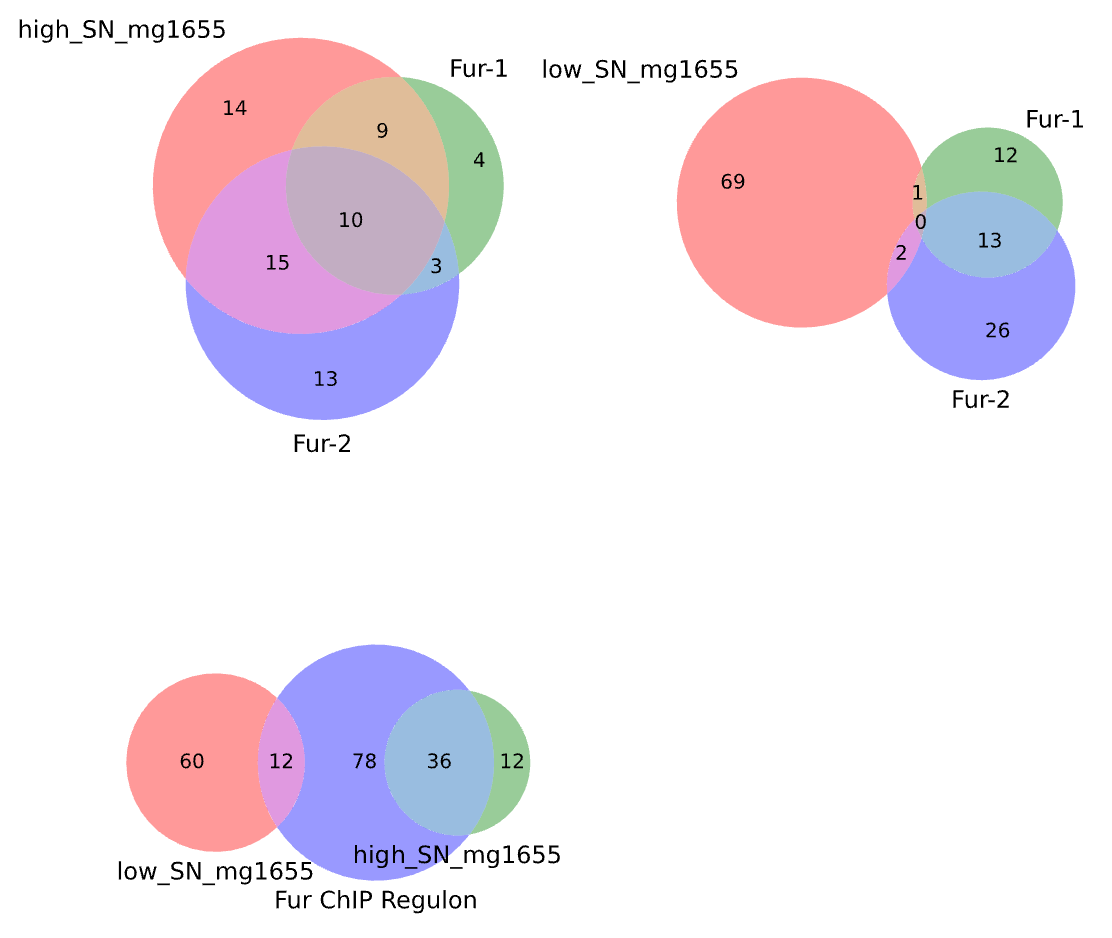


**Fig G.** Venn diagrams showing relationships of genes with high/low S/N ratios and Fur ICA/ChIP regulons for MG1655 strain.


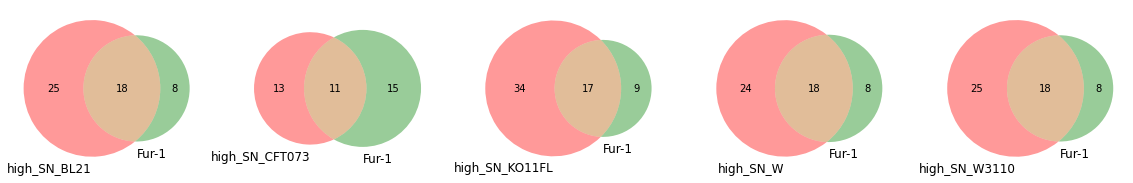


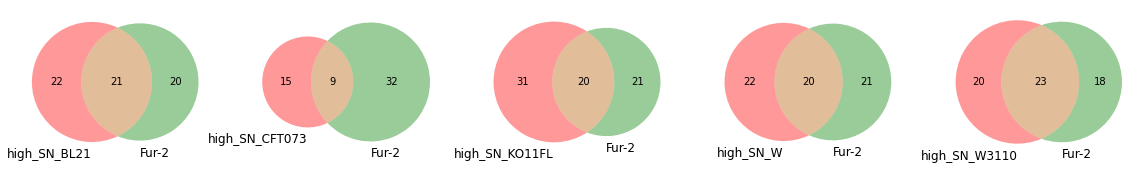


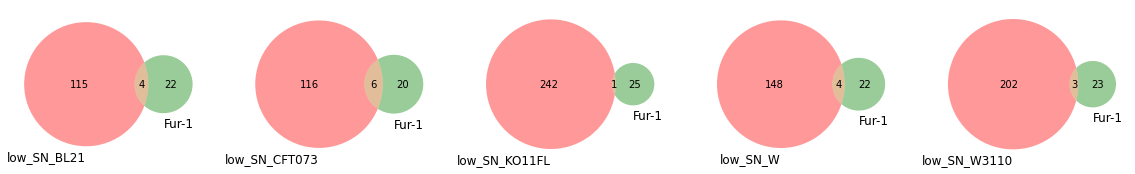


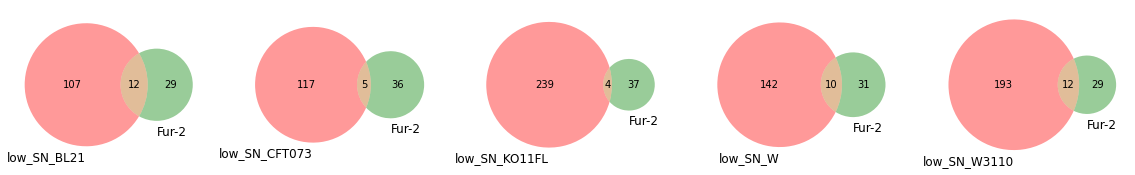


**Fig H.** Venn diagrams of Fur ICA regulons and Fur high/low SN ratio groups across multiple strains: BL21, CFT073, KO11FL, W, and W3110.
